# HPV16 E7 oncoprotein test as a triage strategy for HPV16-positive women in cervical cancer screening: long-term follow-up outcome

**DOI:** 10.3389/fonc.2023.1221962

**Published:** 2023-09-15

**Authors:** Xinmei Wang, Guangnan Shuai, Junhui Xu, Meihua Liu, Jianguo Zhao, Na Zhang, Wenwen Zhang, Pengpeng Qu

**Affiliations:** ^1^ Department of Gynecological Oncology, Tianjin Central Hospital of Gynecology and Obstetrics, Tianjin, China; ^2^ Wenjiang District People’s Hospital of Chengdu, Chengdu, Sichuan, China; ^3^ Tianjin Medical University, Tianjin, China

**Keywords:** cervical cancer, colposcopy, human papillomavirus, E7 oncoprotein, high-grade cervical squamous intraepithelial lesions, ThinPrep cytology test

## Abstract

**Background:**

Colposcopy is recommended once human papillomavirus (HPV)16/18 infection is detected. However, not all HPV16/18-positive women will necessarily develop cervical lesions. Therefore, this study aimed to investigate the application of quantitative HPV16 E7 oncoprotein detection as a cervical cancer screening method for more efficient screening while minimizing unnecessary colposcopy.

**Methods:**

E7 oncoprotein (HPV16) was quantitatively detected in cervical exfoliated cells of HPV16-positive women. The levels of HPV16 E7 oncoprotein in different degrees of cervical lesions were compared, and the optimal cut-off value for identifying HSIL+ was determined by receiver operating characteristic (ROC) curve analysis. With a pathological diagnosis as the gold standard, the sensitivity (SEN), specificity (SPE), positive predictive value (PPV), negative predictive value (NPV), and Kappa value were calculated to verify the diagnostic value of the method. Women diagnosed with low-grade squamous intraepithelial lesions (LSIL) and normal women were followed up for 5 years to evaluate the predictive value of HPV16 E7 protein for disease progression/persistent infection.

**Results:**

The expression level of HPV16 E7 oncoprotein was positively correlated with the degree of the cervical lesion (r = 0.589, P < 0.01). The area under the ROC curve (AUC) was 0.817 (confidence interval: 0.729–0.904). The cut-off value of E7 oncoprotein for identifying HSIL+ was 8.68 ng/ml. The SEN, SPE, PPV, NPV, and Kappa values of HPV16 E7 oncoprotein for the identification of HSIL+ were 87.1%,70.0%, 87.1%, 70.0%, and 0.571, respectively, which were higher than those of ThinPrep cytology test (TCT). The SEN, SPE, PPV, and NPV of HPV16 E7 oncoprotein in predicting disease progression/persistent infection were 93.75%, 91.30%, 88.24%, and 95.45%, respectively.

**Conclusion:**

The quantitative detection of HPV 16 E7 oncoprotein can not only accurately screen cervical lesions but also achieve efficient colposcopy referral. Additionally, HPV16 E7 oncoprotein can accurately predict the progression of cervical lesions and persistent HPV infection.

## 1 Introduction

Cervical cancer is one of the most common malignant tumors among women. In 2020, there were 604,127 new cases of cervical cancer and 341,831 deaths from cervical cancer worldwide (1). A large number of epidemiological and molecular biological studies have confirmed that persistent high-risk HPV infection is closely related to cervical precancerous lesions and cervical cancer (2, 3). For HPV, it takes about 10 years from cervical infection to precancerous lesions and cervical cancer. Therefore, early diagnosis and treatment of HSIL are key to preventing the occurrence of cervical cancer. In 2014, the WHO used the HPV test as a primary screening method for cervical cancer screening (4). Epidemiological studies have shown that infection rates of the virus can be as high as 80% in sexually active women (5). While most viral infections are automatically cleared by the host immune system, very few persist and eventually lead to cancer (6). Worldwide, HPV16 is the dominant type causing invasive cervical cancer (46-63%), followed by HPV18 (10-15%), with an infection preference for HPV16 (2.5%) and HPV18 (0.9%) in women with normal cervical cytology (7).

In recent years, studies have confirmed that HPV E6 and E7 oncoproteins play a key role in viral proliferation and squamous intraepithelial lesions of the cervix until cervical invasive carcinoma is developed (3, 8). Multiple studies have shown that HPV variants differ significantly in the risk of persistent HPV infection and progression to cervical intraepithelial neoplasia (CIN) and CC (9). The overexpression of HPV16 E6 oncoprotein variants in keratinocyte primary cultures and found differences in their ability to induce serum/calcium-resistant colonies and p53/Bax downregulation (10), affecting several important cellular processes, including differentiation, apoptosis (11), immortalization, transformation (12), migration and transfer (13). Studies have shown that in the process of cervical cancer development, HPV E7 oncoprotein was highly expressed in the early stage of precancerous lesions, and its level gradually increased with the aggravation of lesions (14, 15). Therefore, the detection of HPV16 E7 oncoprotein can help identify cervical precancerous lesions.

In this study, HPV16 E7 oncoprotein concentration in cervical exfoliated cells of the normal cervix, LSIL, HSIL, and cervical cancer of HPV16-positive women was detected by using E7 Oncoprotein (HPV16) Diagnostic Kit (Magnetic Particle Chemiluminescence Method). The results of the colposcopic cervical biopsy were used as the gold standard and compared with TCT to evaluate the application value of HPV16 E7 oncoprotein in screening for cervical lesions to explore the risk assessment of the prognosis of cervical lesions caused by HPV16 infection and possible triage strategies.

## 2 Materials and methods

### 2.1 Subjects

A total of 200 HPV16-positive women aged 20–72 years, with an average age of 42.18 years, were selected from the colposcopy clinic of Tianjin Central Hospital of Gynecology and Obstetrics from December 2016 to March 2018.

Inclusion criteria were (i) patients having a sexual life history, with performed TCT test and colposcopy, and cervical histopathological results and who are HPV 16-positive by HPV DNA testing; (ii) non-pregnant patients; (iii) patients without recent vaginal infection and medication; (iv) patients without a history of cervical surgery; (v) patients without a history of cervical cancer and pelvic radiotherapy; (vi) patients who agreed to participate in the study and signed an informed consent form. Patients who did not meet the above conditions were excluded.

Each patient provided a signed copy of informed consent. All procedures performed in studies involving human participants were approved by the Ethics Committee of the Tianjin Central Hospital of Gynecology and Obstetrics (2015KY031).

### 2.2 Research design

A total of 200 women were divided into two parts, of which 100 women were divided into the following four groups based on the pathological results: normal cervix group, LSIL group, HSIL group, and cervical cancer group. The levels of HPV16 E7 oncoprotein among four groups were compared. Spearman correlation analysis was performed to determine the relationship between HPV16 E7 oncoprotein level and cervical lesions. The receiver operating characteristic (ROC) curve was used to determine the accuracy of HPV16 E7 oncoprotein levels in detecting HSIL+, and the cut-off value of HPV16 E7 oncoprotein level was calculated.

Another 100 women were divided into two groups based on cut-off values. TCT was performed simultaneously in these 100 women. Using pathological examination as the gold standard, the SEN, SPE, PPV, NPV, and Kappa values of the two methods were calculated and compared.

**Figure 1:**
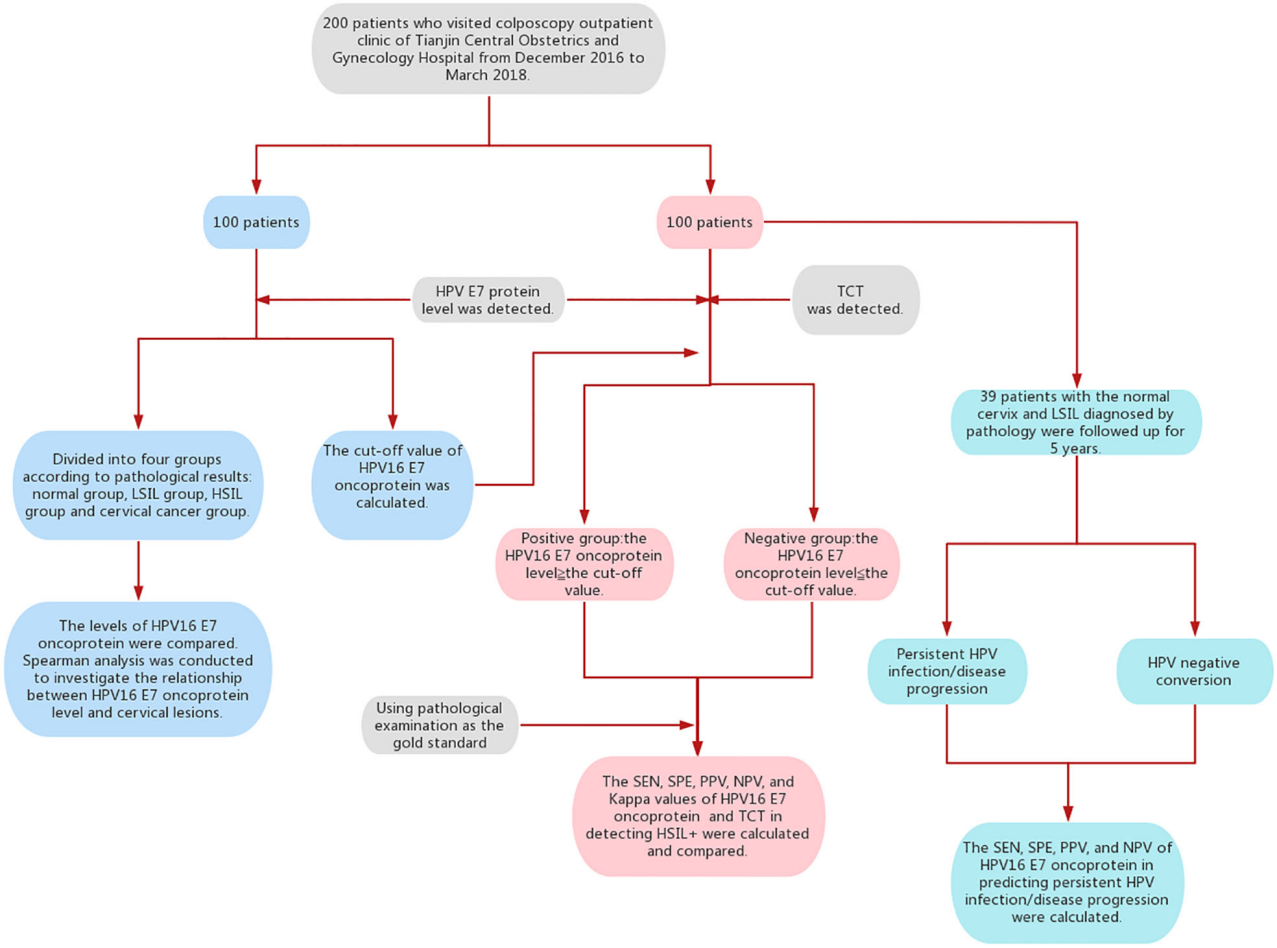
Flowchart of this study.

In addition, 39 patients with the normal cervix and LSIL diagnosed by pathology were followed up for 5 years. Follow-up events included HPV-negative conversion, persistent HPV infection, and disease progression. The endpoint of follow-up was HPV-negative or higher-grade lesions. According to the results of E7 oncoprotein, the patients were divided into a positive group and a negative group. The predictive value of HPV16 E7 oncoprotein for disease progression or persistent HPV infection was analyzed (**Figure 1**).

### 2.3 Sample collection and testing methods

For specimen collection, the mucus and secretions on the cervical surface were wiped with a dry cotton ball. A special cervical brush was used to collect the exfoliated cervical cells at the squamocolumnar junction of the cervix. Brush heads were stored in cell preservation solution(54%methanol) for TCT and freeze-dried tubes forHPV16 E7 oncoprotein detection.

#### 2.3.1 E7 Oncoprotein

The cervical exfoliated cells were collected by rotating a special cervical brush at least five times in the clockwise direction at the junction of the squamous and columnar epithelium of the cervix of the patient. After removing the cervical brush, the brush head was stored in a dry-frozen tube, and the tube was labeled for later use. Specimens were sent to the laboratory for HPV E7 oncoprotein detection within 2 h after collection. Specimens that cannot be tested on the same day were frozen at –20°C and tested within two weeks. HPV16 E7 oncoprotein was detected by magnetic particle-based chemiluminescence immunoassay and automatic chemiluminescence instrument. The HPV 16 E7 oncoprotein in the sample was combined with alkaline phosphatase (AP) labeled monoclonal antibody and fluorescein isothiocyanate (FITC) labeled monoclonal antibody to form antibody-antigen-antibody “sandwich” complex. Then the magnetic particle reagent with an anti-FITC antibody was added to bind the above immune complex to magnetic particles through the specific binding of the anti-FITC antibody to FITC. Under the action of the external magnetic field, the immune complex was separated from other substances, and the enzyme-catalyzed chemiluminescence substrate was added after cleaning the complex. The substrate was catalyzed under the action of the enzyme to form an unstable excitation intermediate. When the exciter returns to the ground state, photons were emitted to form a luminescence reaction. The chemiluminescence instrument was used to detect the luminescence intensity of the reaction. In the monitoring range, the luminous intensity was proportional to the content of HPV E7 oncoprotein in the sample, and the concentration of E7 oncoprotein in the sample was calculated by using the improved four-parameter Logistic equation. The experimental procedures were conducted strictly following the operating instructions of E7 Oncoprotein (HPV16) Diagnostic Kit (Magnetic Particle Chemiluminescence Method) and CIA 1200 Automatic Magnetic Particle Chemiluminescence Immunoassay Analyzer from FAMID Biomedical Technology (Tianjin) Co., Ltd.

#### 2.3.2 TCT examination

TCT examination was performed as follows. Cytological slides were prepared using the ThinPrep2000 system. The samples were pretreated with digestive fluid (glacial acetic acid: cleaning fluid = 1:9) and programmed with ThinPrep machine (Hologic, USA), followed by fixed staining. Results were reported using the Bethesda Cervical Cytology Reporting System (TBS). They were divided as negative for intraepithelial lesion or malignancy (NILM), atypical squamous cell of undetermined significance (ASCUS), ASC-H (atypical squamous cells cannot exclude HSIL), LSIL, HSIL, and atypical glandular cell (AGC).

A colposcopy specialist performed a colposcopy and biopsy. The cervical tissues were analyzed and diagnosed by at least two pathologists.

### 2.4 Statistical analysis

Statistical analysis was performed using SPSS28.0 software. Analysis of variance was used to compare the differences in E7 oncoprotein levels among the groups. Spearman correlation analysis was used to analyze the relationship between E7 oncoprotein and the degree of cervical lesions. The ROC curve was used to determine the cut-off value of HPV16 E7 oncoprotein levels in detecting HSIL+. SEN, SPE, PPV, NPV, and LR were calculated to evaluate the diagnostic performance. The Kappa test was used to evaluate the consistency of E7 oncoprotein and TCT methods and pathological diagnosis. All tests were two-sided, and a p-value < 0.05 was considered statistically significant.

## 3 Results

### 3.1 Relationship between HPV16 E7 oncoprotein and cervical lesions

According to the pathological results, 100 patients were divided into four groups: normal cervix group (n = 21), LSIL group (n = 9), HSIL group (n = 65), and cervical cancer group (n = 5). HPV16 E7 oncoprotein concentration was significantly different among the four groups (P < 0.001) (**Table 1**). The Spearman correlation analysis showed that the expression level of HPV16 E7 oncoprotein was positively correlated with the degree of cervical lesions. The more serious the degree of cervical lesions, the higher the expression levels were (r = 0.589, P < 0.01).

**Table 1:** Comparison of HPV16 E7 oncoprotein levels in different degrees of cervical lesions.

Group	N	HPV16 E7 Oncoprotein(ng/ml)*
normal cervix	21	4.34 (0.5–17.81)
LSIL	9	5.67 (2.46–25.25)
HSIL	65	43.05 (17.09–170.02)
CA	5	25.9 (17.36–399.43)
P Value	<0.001	

*Median of HPV 16 E7 oncoprotein concentration, with 25th–75th percentile in parentheses.

### 3.2 Diagnostic accuracy of HPV16 E7 oncoprotein in detecting HSIL+

**Figure 2:**
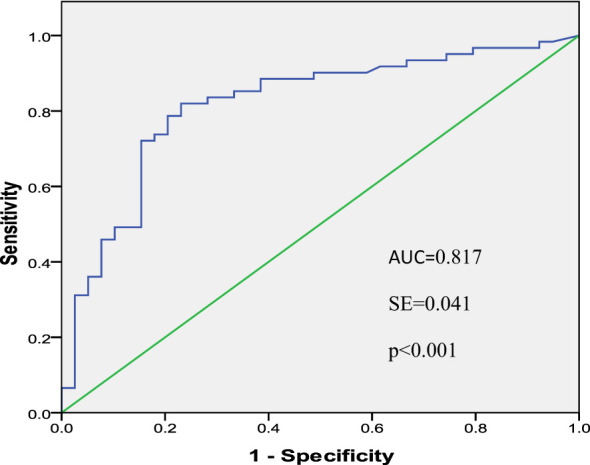
ROC Curve of HPV16 E7 oncoprotein in detecting HSIL+.

A total of 100 women were divided into two groups according to pathology. (1) The control group: the pathological diagnosis was normal cervix and normal LSIL (n = 30). (2) The observation group: the pathological diagnosis was HSIL and cervical cancer (n = 70). The ROC curve was obtained to evaluate the accuracy of HPV16 E7 oncoprotein in detecting HSIL+ (**Figure 2**). The area under the ROC curve (AUC) was 0.817 (confidence interval: 0.729–0.904). The diagnostic detection threshold of HPV16 E7 was ≥ 8.68 ng/ml.

### 3.3 Diagnostic performance of HPV16 E7 oncoprotein in detecting HSIL+

According to pathological findings, normal cervix and LSIL were negative, and HSIL and cervical cancer were positive. Another 100 women were divided into two groups based on HPV16 E7 oncoprotein levels: (1) the positive group, in which E7 oncoprotein level was ≥ 8.68 ng/ml (n = 70), and (2) the negative group, in which E7 oncoprotein level was < 8.68 ng/ml (n = 30). Furthermore, 100 women were divided into two groups based on TCT results: (1) the positive group comprising ASCUS, ASC-H, LSIL, HSIL, and cervical cancer (n = 64) and (2) the negative group comprising NILM (n = 36). The results showed that the SEN, SPE, PPV, and NPV of HPV16 E7 oncoprotein in detecting HSIL+ were 87.1%, 70.0%, 87.1%, and 70.0%, respectively, which were higher than those of TCT. Taking pathological diagnosis as the gold standard, the results showed that the Kappa value of HPV16 E7 oncoprotein was 0.571 compared to 0.369 by TCT (**Table 2**).

**Table 2:** Diagnostic performance of HPV16 E7 oncoprotein in detecting HSIL+.

Detection method		Pathology		SEN(%)	SPE(%)	PPV(%)	NPV(%)	Kappa value
		+	-					
E7	+	61	9	87.1	70.0	87.1	70.0	0.571
	-	9	21					
TCT	+	53	11	75.7	63.3	82.8	52.8	0.369
	-	17	19					

### 3.4 Predictive value of HPV16 E7 oncoprotein for disease progression and persistent HPV infection

Thirty-nine patients with the normal cervix and LSIL in part 1 were followed up for 5 years. Among them, 31 had normal cervix, and 8 had LSIL. All patients were divided into negative and positive groups according to the cut-off value of HPV16 E7 oncoprotein. The proportion of HPV negative conversion in the positive group was significantly lower than that in the negative group, and the proportion of persistent infection and disease progression in the positive group was significantly higher than that in the negative group (P = 0.001) (**Table 3**). The SEN, SPE, PPV, and NPV of HPV16 E7 oncoprotein in predicting disease progression/persistent infection were 93.75%, 91.30%, 88.24%, and 95.45%, respectively (**Table 4**).

**Table 3:** Comparison of outcomes in patients with different levels of HPV16 E7 oncoprotein.

Group	N (%)	HPV Negative conversion (%)	HPV Persistent Infection (%)	Disease Progression (%)
Negative group	22 (56.4)	16 (72.7)	5 (22.7)	1 (4.5)
Positive group	17 (43.6)	3 (17.6)	5 (29.5)	9 (43.6)
Total	39	19 (48.7)	10 (25.6)	10 (25.6)
*Chi-*square	14.899			
P Value	0.001			

**Table 4:** Predictive value of HPV16 E7 oncoprotein for disease progression and persistent HPV infection.

Outcome of Patients	E7 Oncoprotein		SEN(%)	SPE(%)	PPV(%)	NPV(%)
	+	-				
HPV Persistent Infection/Disease Progression	15	1	93.75	91.30	88.24	95.45
HPV Negative conversion	2	21				

## 4 Discussion

HPV infection, especially high-risk HPV infection, is closely related to cervical precancerous lesions and cervical cancer, and about 99.7% of cervical cancer patients have an HPV infection (2). The most common type of HPV infection in cervical cancer is HPV16 (16–18). At present, HPV testing replaces cytology for cervical cancer screening because of greater sensitivity and superior reassurance following negative tests. The management of women who tested positive is still unresolved. Once the woman is HPV16/18-positive, colposcopy is suggested. However, only a small percentage of HPV16-positive women will develop HSIL. A 7-year follow-up study of 11,573 HPV-positive women has found that 26% of the population were HPV16-positive, and 22% of HPV16-positive women progressed to CIN3+ (19). The outcome of most HPV infections detected in screening is evident within three years, when the vast majority of infections have been cleared (20). The progression from HSIL to invasive cervical cancer is a long process, usually taking 3 to 8 years (21). Thus, a large number of HPV16-positive women continue to undergo unnecessary colposcopy. Therefore, new triage methods and prognostic risk assessments are necessary, even for women who are HPV16-positive.

At present, the detection methods of HPV primarily use RNA or DNA, and there are few detection methods for oncoproteins. However, studies have confirmed that after cervical epithelial cells are infected with HPV, the expression of E6/E7 oncoprotein leads to the development of cervical cancer (22, 23). Additionally, the advanced stage of cervical cancer is mainly associated with E6 oncoprotein, while the early cancer stage is closely related to E7 oncoprotein (24). The increased levels of E6/E7 oncoprotein are important for inducing the transformation and carcinogenesis of cervical epithelial cells (25). Therefore, detecting HPV E6/E7 expression products to determine the carcinogenic activity of HPV and the risk of cervical cancer has garnered increasing attention in recent years. Presently, several commercial HPV E6/E7 mRNA assays are available with gradually recognized clinical value. However, only a few quantitative methods can detect HPV E6/E7 oncoprotein. Therefore, we selected HPV16 E7 oncoprotein as a new marker for cervical cancer screening.

In this study, the magnetic particle chemiluminescence method could quantitatively detect HPV16 E7 oncoprotein levels in cervical exfoliated cells. The correlation analysis of HPV16 E7 oncoprotein levels and the severity of cervical lesions was performed in 100 cases. The results showed that the expression of HPV16 E7 oncoprotein was positively correlated with the degree of cervical lesions, which is consistent with past research results (15, 26). The results indicated that the detection of HPV16 E7 oncoprotein could objectively reflect the degree of cervical precancerous lesions and also suggested the possibility of the quantitative detection of HPV16 E7 oncoprotein as a new method of cervical cancer screening.

Previous studies have confirmed that HPV DNA detection has high sensitivity and insufficient specificity when screening for HSIL, while TCT screening has high specificity but significantly reduced sensitivity, resulting in a partially missed diagnosis (27, 28). At the same time, studies have shown that the HPV E7 mRNA assay had a higher specificity and positive predictive value than the HPV DNA assay and, thus, has a higher diagnostic value for the diagnosis of HSIL and cervical cancer, with a sensitivity of 91%–95% and specificity of 42%–61% (29–31). Furthermore, Shi et al. have detected HPV E6/E7 oncoprotein in cervical biopsies by western blot and shown that HPV E6/E7 oncoprotein had better sensitivity in diagnosing HISL than TCT detection and better specificity than HPV E6/E7 mRNA and HPV DNA detection (23).

In this study, the results showed that HPV16 E7 oncoprotein could accurately distinguish normal cervix and LSIL from HSIL and cervical cancer (AUC = 0.817, P < 0.001). Compared with TCT detection, the E7 oncoprotein assay used in this study had a higher SPE, PPV (87.1% *vs.* 82.2%), SEN (87.1% *vs.* 75.7%), and NPV (70.0% *vs.* 52.8%). The results suggested that HPV16 E7 oncoprotein detection could not only accurately screen for abnormal cervical lesions but also accurately identify LSIL and normal cervix to reduce unnecessary colposcopy. It is expected to become a new indicator of cervical precancerous lesions and cervical cancer screening.

About 60% of LSIL patients will naturally subside, only needing close observations of the follow-up patients. However, about 20% of HSIL will continue to progress, and 5% will develop into invasive cervical cancer (2). Therefore, all patients with HSIL and a higher degree of cervical lesions need to be treated. Looking for potential biomarkers can help us identify patients with a high risk of disease progression, and early intervention for these patients can effectively reduce the incidence of cervical cancer. HPV16 E7 oncoprotein can effectively distinguish HPV16 transient infection and persistent infection, which confirms the accuracy of E7 oncoprotein in predicting the prognosis risk of patients.

In this study, it was found that the SEN, SPE, PPV, and NPV of HPV16 E7 oncoprotein in predicting disease progression/persistent infection were 93.75%, 91.30%, 88.24%, and 95.45%, respectively. The results suggested that E7 oncoprotein detection has an excellent diagnostic effect in predicting disease progression and persistent HPV infection. For cases with positive E7 oncoprotein, it is necessary to strengthen the screening intensity or even give active treatment. For cases with negative E7 oncoprotein, the interval between tests can be lengthened as appropriate to avoid unnecessary colposcopy or treatment.

## 5 Limitations

This study only tested E7 oncoprotein of HPV16 in a single center. Future large-scale studies need to expand HPV types and include multiple centers to evaluate the value of HPV E7 oncoprotein in cervical cancer screening in HPV-positive women.

## 6 Conclusion

The HPV16 E7 oncoprotein detection method—E7 Oncoprotein (HPV16) Diagnostic Kit (Magnetic Particle Chemiluminescence Method)—used in this study was accurate, reliable, and easy to perform. Compared with TCT, HPV16 E7 oncoprotein detection had better diagnostic value for HSIL+ and could be used for efficient colposcopy referral. In addition, it also had a good predictive value for the prognosis of LSIL and lower-grade lesions. It is expected to be widely used for the clinical detection of cervical lesions and become a new indicator of precancerous cervical lesions and cervical cancer screening.

## Data availability statement

The original contributions presented in the study are included in the article/supplementary material, further inquiries can be directed to the corresponding author/s.

## Ethics statement

The studies involving humans were approved by Ethics Committee of the Tianjin Central Hospital of Gynecology and Obstetrics (2015KY031). The studies were conducted in accordance with the local legislation and institutional requirements. The participants provided their written informed consent to participate in this study.

## Author contributions

PQ: project development, laboratory work, and manuscript revision. XW: data collection, statistical processing, and manuscript writing. GS: experimental process, data collection, statistical processing, and manuscript writing. JX: experimental process, data collection, and statistical processing. ML: sample and data collection. JZ: sample and data collection. NZ: sample and data collection. WZ: laboratory work and manuscript revision. All authors contributed to the article and approved the submitted version.

## References

[1] Sung H Ferlay J Siegel RL Laversanne M Soerjomataram I Jemal A. Global Cancer Statistics 2020: GLOBOCAN estimates of incidence and mortality worldwide for 36 cancers in 185 countries. CA Cancer J Clin (2021) 71(3):209–49. doi: 10.3322/caac.21660 33538338

[2] de Sanjosé S Brotons M Pavón MA. The natural history of human papillomavirus infection. Best Pract Res Clin Obstet Gynaecol (2018) 47:2–13. doi: 10.1016/j.bpobgyn.2017.08.015 28964706

[3] Liu M Yan X Zhang M Li X Li S Jing M. Influence of Human papillomavirus infection on the natural history of cervical intraepithelial neoplasia 1: a meta-analysis. BioMed Res Int (2017) 2017:8971059. doi: 10.1155/2017/8971059 28812024 PMC5546131

[4] World Health Organization. Comprehensive Cervical Cancer Control: A Guide to Essential Practice. Geneva: World Health Organization (2014). 25642554

[5] Chesson HW Dunne EF Hariri S Markowitz LE. The estimated lifetime probability of acquiring human papillomavirus in the United States. Sex Transm Dis (2014) 41:660–4. doi: 10.1097/OLQ.0000000000000193 PMC6745688 25299412

[6] Winer RL Hughes JP Feng Q Xi LF Cherne S O’Reilly S. Early natural history of incident, type-specific human papillomavirus infections in newly sexually active young women. Cancer Epidemiol Biomarkers Prev (2011) 20:699–707. doi: 10.1158/1055-9965.EPI-10-1108 21173170 PMC3078690

[7] de Sanjosé S Diaz M Castellsagué X Clifford G Bruni L Muñoz N. Worldwide prevalence and genotype distribution of cervical human papillomavirus DNA in women with normal cytology: a meta-analysis. Lancet Infect Dis (2007) 7:453– 9. doi: 10.1016/S1473-3099(07)70158-5 17597569

[8] McBride AA Warburton A. The role of integration in oncogenic progression of HPV-associated cancers. PloS Pathog (2017) 13(4):e1006211. doi: 10.1371/journal.ppat.1006211 28384274 PMC5383336

[9] Freitas LB Chen Z Muqui EF Boldrini NA MIranda AE Spano LC. Human papillomavirus 16 non-European variants are preferentially associated with high-grade cervical lesions. PloS One (2014) 9:e100746. doi: 10.1371/journal.pone.0100746 24983739 PMC4077691

[10] Asadurian Y Kurilin H Lichtig H Jackman A Gonen P Tommasino M. Activities of human papillomavirus 16 E6 natural variants in human keratinocytes. J Med Virol (2007) 79:1751–60. doi: 10.1002/jmv.20978 17854024

[11] Zehbe I Lichtig H Westerback A Lambert PF Tommasino M Sherman L. Rare human papillomavirus 16 E6 variants reveal significant oncogenic potential. Mol Cancer (2011) 10:77. doi: 10.1186/1476-4598-10-77 21702904 PMC3144020

[12] Richard C Lanner C Naryzhny SN Sherman L Lee H Lambert PF. The immortalizing and transforming ability of two common human papillomavirus 16 E6 variants with different prevalences in cervical cancer. Oncogene (2010) 29:3435–45. doi: 10.1038/onc.2010.93 20383192

[13] Niccoli S Abraham S Richard C Zehbe I. The Asian-American E6 variant protein of human papillomavirus 16 alone is sufficient to promote immortalization, transformation, and migration of primary human foreskin keratinocytes. J Virol (2012) 86:12384–96. doi: 10.1128/JVI.01512-12 PMC3486486 22951839

[14] Yoshimatsu Y Nakahara T Tanaka K Inagawa Y Narisawa-Saito M Yugawa T. Roles of the PDZ-binding motif of HPV 16 E6 protein in oncogenic transformation of human cervical keratinocytes. Cancer Sci (2017) 108(7):1303–9. doi: 10.1111/cas.13264 PMC5497797 28440909

[15] Zhang JJ Cao XC Zheng XY Wang HY Li YW. Feasibility study of a human papillomavirus E6 and E7 oncoprotein test for the diagnosis of cervical precancer and cancer. J Int Med Res (2018) 46(3):1033–42. doi: 10.1177/0300060517736913 PMC5972251 29322839

[16] Kombe AJ Li B Zahid A Mengist HM Bounda GA Zhou Y. Epidemiology and burden of human papillomavirus and related diseases, molecular pathogenesis, and vaccine evaluation. Front Public Health (2021) 8:552028. doi: 10.3389/fpubh.2020.552028 33553082 PMC7855977

[17] Graham SV. The human papillomavirus replication cycle, and its links to cancer progression: a comprehensive review. Clin Sci (Lond) (2017) 131(17):2201–21. doi: 10.1042/CS20160786 28798073

[18] Wu P Xiong H Yang M Li L Wu P Lazare C. Co-infections of HPV16/18 with other high-risk HPV types and the risk of cervical carcinogenesis: A large population-based study. Gynecol Oncol (2019) 155(3):436–43. doi: 10.1016/j.ygyno.2019.10.003 31604662

[19] Demarco M Hyun N Carter-Pokras O Raine-Bennett TR Cheung L Chen X. A study of type-specific HPV natural history and implications for contemporary cervical cancer screening programs. EClinicalMedicine (2020) 22:100293. doi: 10.1016/j.eclinm.2020.100293 32510043 PMC7264956

[20] Plummer M Schiffman M Castle PE Maucort-Boulch D Wheeler CM. Group A. A 2-year prospective study of human papillomavirus persistence among women with a cytological diagnosis of atypical squamous cells of undetermined significance or low-grade squamous intraepithelial lesion. J Infect Dis (2007) 195(11):1582–9. doi: 10.1086/516784 17471427

[21] Hoppenot C Stampler K Dunton C. Cervical cancer screening in high- and low-resource countries: implications and new developments. Obstet Gynecol Surv (2012) 67(10):658–67. doi: 10.1097/OGX.0b013e3182732375 23112073

[22] Pal A Kundu R. Human papillomavirus E6 and E7: the cervical cancer hallmarks and targets for therapy. Front Microbiol (2019) 10:3116. doi: 10.3389/fmicb.2019.03116 32038557 PMC6985034

[23] Shi WJ Liu H Wu D Tang ZH Shen YC Guo L. E6/E7 proteins are potential markers for the screening and diagnosis of cervical precancerous lesions and cervical cancer in a Chinese population. Oncol Lett (2017) 14(5):6251–8. doi: 10.3892/ol.2017.6932 PMC5661417 29113275

[24] Song S Pitot HC Lambert PF. The human papillomavirus type 16 E6 gene alone is sufficient to induce carcinomas in transgenic animals. J Virol (1999) 73(7):5887–93. doi: 10.1128/JVI.73.7.5887-5893.1999 PMC112649 10364340

[25] Ghittoni R Accardi R Hasan U Gheit T Sylla B Tommasino M. The biological properties of E6 and E7 oncoproteins from human papillomaviruses. Virus Genes (2010) 40(1):1–13. doi: 10.1007/s11262-009-0412-8 19838783

[26] Agorastos T Chatzistamatiou K Moysiadis T Kaufmann AM Skenderi A Lekka I. Human papillomavirus E7 protein detection as a method of triage to colposcopy of HPV positive women, in comparison to genotyping and cytology. Final results of the PIPAVIR study. Int J Cancer (2017) 141(3):519–30. doi: 10.1002/ijc.30761 28470689

[27] Liu Y Zhang L Zhao G Che L Zhang H Fang J. The clinical research of Thinprep Cytology Test (TCT) combined with HPV-DNA detection in screening cervical cancer. Cell Mol Biol (Noisy-le-grand) (2017) 63(2):92–5. doi: 10.14715/cmb/2017.63.2.14 28364788

[28] Sawaya GF Smith-McCune K Kuppermann M. Cervical cancer screening: more choices in 2019. JAMA (2019) 321(20):2018–9. doi: 10.1001/jama.2019.4595 PMC6656358 31135834

[29] Tisi G Gargiulo F Gozzini E Baronchelli C Odicino F Salinaro F. Role of HPV DNA, HPV mRNA and cytology in the follow-up of women treated for cervical dysplasia. APMIS (2019) 127(4):196–201. doi: 10.1111/apm.12931 30815926

[30] Giorgi Rossi P Carozzi F Ronco G. p16/ki67 and E6/E7 mRNA accuracy and prognostic value in triaging HPV DNA-positive women. J Natl Cancer Inst (2021) 113(3):292–300. doi: 10.1093/jnci/djaa105 32745170 PMC7936054

[31] Derbie A Mekonnen D Woldeamanuel Y Van Ostade X Abebe T. HPV E6/E7 mRNA test for the detection of high grade cervical intraepithelial neoplasia (CIN2+): a systematic review. Infect Agent Cancer (2020) 15:9. doi: 10.1186/s13027-020-0278-x 32047531 PMC7006188

